# Origin of the Rare Hybrid Genus ×*Trisetokoeleria* Tzvelev (*Poaceae*) According to Molecular Phylogenetic Data

**DOI:** 10.3390/plants11243533

**Published:** 2022-12-15

**Authors:** Alexander A. Gnutikov, Nikolai N. Nosov, Tatiana M. Koroleva, Elizaveta O. Punina, Nina S. Probatova, Victoria S. Shneyer, Alexander V. Rodionov

**Affiliations:** 1 Department of Genetic Resources of Oat, Barley, Rye, Federal Research Center N. I. Vavilov All-Russian Institute of Plant Genetic Resources (VIR), 190000 St. Petersburg, Russia; 2 Laboratory of Biosystematics and Cytology, Komarov Botanical Institute of the Russian Academy of Sciences, 197376 St. Petersburg, Russia; 3 Laboratory of Geography and Vegetation Mapping, Komarov Botanical Institute of the Russian Academy of Sciences, 197376 St. Petersburg, Russia; 4 Laboratory of Botany, Federal Scientific Center of the East Asia Terrestrial Biodiversity, Far Eastern Branch of the Russian Academy of Sciences, 690022 Vladivostok, Russia

**Keywords:** rRNA genes, grasses, hybridization, ITS, NGS, phylogeny, Poeae, *trn*K–*rps*16, *trn*L–*trn*F

## Abstract

In our article, we analyzed new data on the origin of the hybrid genus ×*Trisetokoeleria*. According to the morphological criteria ×*T. jurtzevii* is a hybrid between *Koeleria asiatica* s. l. and *Trisetum spicatum*, ×*T. taimyrica*, and originated from *Koeleria asiatica* s. l. and *Trisetum subalpestre*, ×*T. gorodkowii*, a hybrid between *Koeleria asiatica* and *Trisetum ruprechtianum*. Later ×*T. taimyrica* was transferred to *Koeleria*. Parental taxa are prone to active hybridization themselves, thus, new methods of next-generation sequencing (NGS) were needed to clarify the relationships of these genera. For NGS we used the fragment 18S rDNA (part)–ITS1–5.8S rDNA (totally 441 accessions). We analyzed ITS1–5.8S rDNA–ITS2 region, *trn*L–*trn*F and *trn*K–*rps*16 from eight samples of the five species, using the Sanger method: ×*Trisetokoeleria jurtzevii*, ×*T. taimyrica*, *Koeleria asiatica*, *Sibirotrisetum sibiricum* (=*Trisetum sibiricum*), and *Trisetum spicatum*. We also studied the pollen fertility of ×*Trisetokoeleria* and its possible progenitors. Our data partly contradicted previous assumptions, based on morphological grounds, and showed us a picture of developed introgression within and between *Koeleria* and *Trisetum*. ×*T. jurtzevii*, a totally sterile hybrid formed rather recently. We can suppose that ×*T. jurtzevii* is a hybrid between *K. asiatica* and some *Trisetum* s. str. Species, but not *T. spicatum*. ×*T. gorodkowii*, a hybrid in the stage of primary stabilization; it has one unique ribotype related to *T. spicatum* s. l. The second parental species is unrelated to *Trisetum ruprechtianum*. ×*T. taimyrica* and is a stabilized hybrid species; it shares major ribotypes with the *T. spicatum*/*T. wrangelense* group and has a minor fraction of rDNA related to genus *Deyeuxia* s. l.

## 1. Introduction

The rare hybrid genus (nothogenus) ×*Trisetokoeleria* Tzvelev of the tribe Poeae s. l. grows in the Arctic regions of Siberia and the Russian Far East. Together with putative parental genera *Koeleria* Pers. and *Trisetum* Pers., it belongs to the subtribe Koeleriinae Asch. et Graebn. [1,2]. The species of ×*Trisetokoeleria* differ from the members of the genus *Koeleria* by having a short (1–3 mm), slightly curved or straight, awn on the lemma placed slightly (0.5–1.5 mm) below its top, semi-transparent glabrous glumes, and short and thick pilose (or scabrous) panicle branches [3]. From the second genus, *Trisetum*, ×*Trisetokoeleria* differs by having a significantly shorter lemma awn [3]. The anthers of ×*Trisetokoeleria* are shorter or longer than those of the parental taxa. The genus comprises three nothospecies. Tzvelev, based on the presence of intermediate morphological characters believed to be those of ×*T. gorodkowii* (Roshev.), was a hybrid between *Koeleria asiatica* Domin s. l. and *Trisetum ruprechtianum* Tzvelev (*T. sibiricum* Rupr. subsp. *litorale* Rupr.) [3]. On the other hand, ×*T. jurtzevii* Prob. was considered a hybrid between *K. asiatica* s. l. and *T. spicatum* (L.) K. Richt. [4], and ×*T. taimyrica* Tzvelev originated from *K. asiatica* s. l. and *Trisetum subalpestre* (Hartm.) Neuman (*T. agrostideum* (Laest.) Fries) [5].

The subtribe Koeleriinae itself has a complex evolutionary history. Previous research established intermingling of the *Trisetum* and *Koeleria* in molecular phylogenetic schemes, as well as separation of some of the *Trisetum* s. l. species within the subtribe [6,7,8,9]. The members of this subtribe, along with all genera of the Aveneae chloroplast group within the Poeae tribe [1,2], are prone to multiple hybridizations.

So, the hybrid nature of the genus ×*Trisetokoeleria* was assumed on the basis of morphological characteristics. However, molecular phylogenetic analysis that could confirm or refute this assumption has not yet been performed. Only one species, ×*T. taimyrica*, was studied in previous molecular phylogenetic analyses and, as a result of this work, the species was assigned to the genus *Koeleria* [8].

Our goal was to find out which species may have participated in the formation of the species of ×*Trisetokoeleria* and whether the DNA sequence data were consistent with morphological observations.

To identify the origin of the hybrid taxa, the ITS region, as well as chloroplast sequences, were mostly used. In our case, we studied the intragenomic variability of the transcribed spacer ITS1 using Illumina high-throughput next-generation sequencing (NGS). This approach is suitable for revealing the hidden polymorphism of hybrid taxa in the case of multiple crossing [10,11,12]. In addition, we took into analysis ITS1–5.8S rDNA–ITS2 sequences of the nuclear genome, as well as *trn*L–*trn*F and *trn*K–*rps*16 sequences of the chloroplast genome, obtained by the Sanger method.

## 2. Results

Marker sequences obtained via NGS comprised 5′–18S rDNA, partial sequence–ITS1–5.8S rDNA, partial sequence. In total, they had 334 nucleotide positions. Our data on herbarium specimens and results of NGS are shown in Table 1, chromosome numbers of the studied species are given according to [13]. After processing, the ITS1 sequences were sorted into variants, so called ribotypes [14,15], each of which corresponded to a single sequence with a certain number of reads per the whole rDNA pool. The major ribotypes (more than 1000 reads per rDNA pool of amplicons) are shown in Table 1.

The sequences obtained in our study by the Sanger method are given in Table 2.

Primary structure of the major ribotypes is given in Table 3.

**Figure 1:**
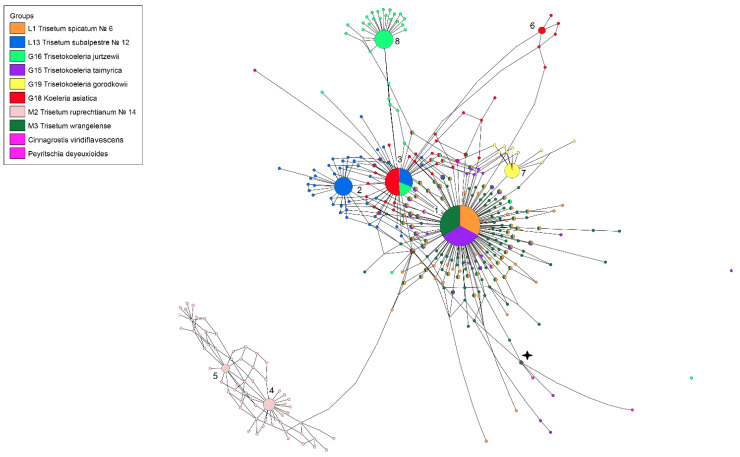
Ribotype network of species of the genus ×*Trisetokoeleria* and putative parental species (NGS data). Radius of the circles on the ribotype network is proportional to the percent number of reads for each ribotype, as shown in the Table 1. Major ribotypes are larger than others and marked with numbers 1–8. The smallest circles correspond to ITS1 variants that have been read fewer than 1000 times.

We observed that the ribotypes had few distinguishing positions (SNPs) but were group-specific. The ribotype network built in TCS 2.1, and visualized in TCS BU, is shown in Figure 1.

**Figure 2:**
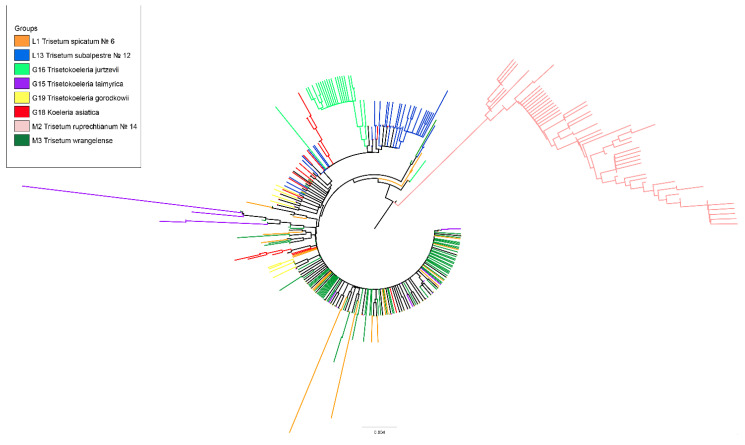
Neighbor-joining tree, based on sequences of 441 accessions of ITS1 ribotypes of the genus ×*Trisetokoeleria* and its probable progenitors, obtained via NGS. The phylogenetic tree was constructed in MEGA X using the pairwise genetic distance matrix, evolutionary model GTR + G, Maximum Composite Likelihood algorithm.

Minor variants and all singletons were derivatives of the major variants (ribotypes) (Figure 1). Table 3 shows two minor ribotypes of ×*Trisetokoeleria taimyrica*, 55 and 14 reads, respectively, which represented a minor fraction in the hybrid genome, probably related to those of the genus *Deyeuxia* Clarion ex P.Beauv. s. l. (incl. *Cinnagrostis* Griseb.). We took as the consensus sequence that of *Trisetum spicatum*, one of the presumed ancestral species of the hybrid ×*Trisetokoeleria*. Major ribotypes were divided into those that were common for different species and those that were species-specific (Figure 1, Table 3). *Trisetum spicatum*, which had the main ribotype, T1, shared this ribotype with both *T. wrangelense* (21,565 reads, 78% per its rDNA pool) and ×*Trisetokoeleria taimyrica* (14,574 reads, 76%). The T1 was the only major ribotype of ×*T. taimyrica.* In addition, the ribotype T1 was present in the genome of *Koeleria asiatica* (1505 reads, 7%). *Trisetum subalpestre*, the possible ancestor of ×*T. taimyrica*, on the contrary, had the unique major ribotype, Ts (12,538 reads, 47%), and the second major ribotype T2 (8685 reads, 33%), also found in the genomes of *Koeleria asiatica* (12,064 reads, 54%) and ×*T. jurtzevii* (2824 reads, 19%). The species *Trisetum ruprechtianum* (=*T. sibiricum* subsp. *litorale*) was only distantly related with the other studied species, and its two major ribotypes, T3 and T4, were unique (Table 1). The ×*Trisetokoeleria gorodkowii* had one major ribotype T5 (3492 reads, 29%). This ribotype also occurred in the minor rDNA fraction of *Trisetum spicatum* and *T. wrangelense* (18 and 12 reads, respectively). The species ×*Trisetokoeleria jurtzevii* had the unique main ribotype Tj (7090 reads, 48%). The neighbor-joining phylogenetic tree (Figure 2) showed almost the same results with an obviously separate clade of *Trisetum ruprechtianum* and ×*Trisetokoeleria jurtzevii* major ribotypes.

**Figure 3:**
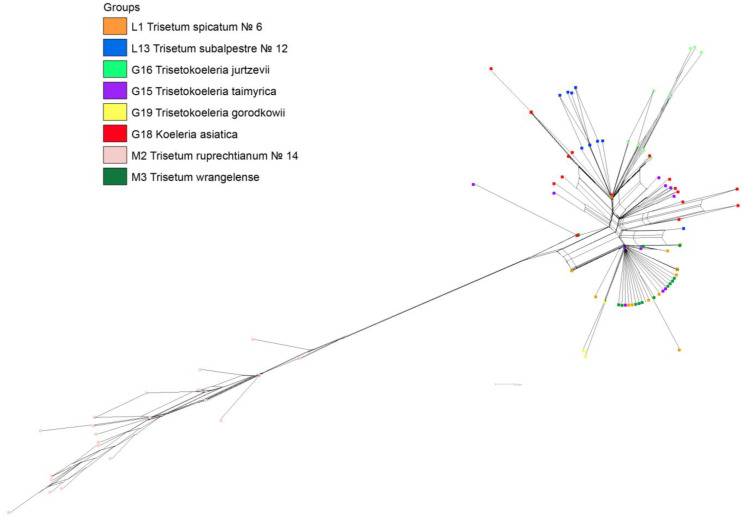
Neighbor-net of the ribotypes of the genus ×*Trisetokoeleria* and putative parents built using SplitsTree v. 4.18.2. For analysis we took ribotypes with the threshold 30 reads per rDNA pool.

The ribotypes of ×*T. taimyrica* were intermingled with those of *Trisetum wrangelense* and *T. spicatum* (Figure 2). The second major ribotype of *Koeleria asiatica* and derivatives of this ribotype formed separate branches, as well as those of *T. wrangelense*. All ribotypes of *Trisetum ruprechtianum* (a member of *T. sibiricum* = *Sibirotrisetum sibiricum* group) belonged to the single group sister of all the other species. The neighbor joining network constructed in SplitsTree 4.18 demonstrated a rougher pattern where the ribotypes of ×*Trisetokoeleria jurtzevii*, ×*T. gorodkowii*, *Trisetum ruprechtianum*, and some ribotypes of *Koeleria asiatica*, were obviously separated (Figure 3).

**Figure 4:**
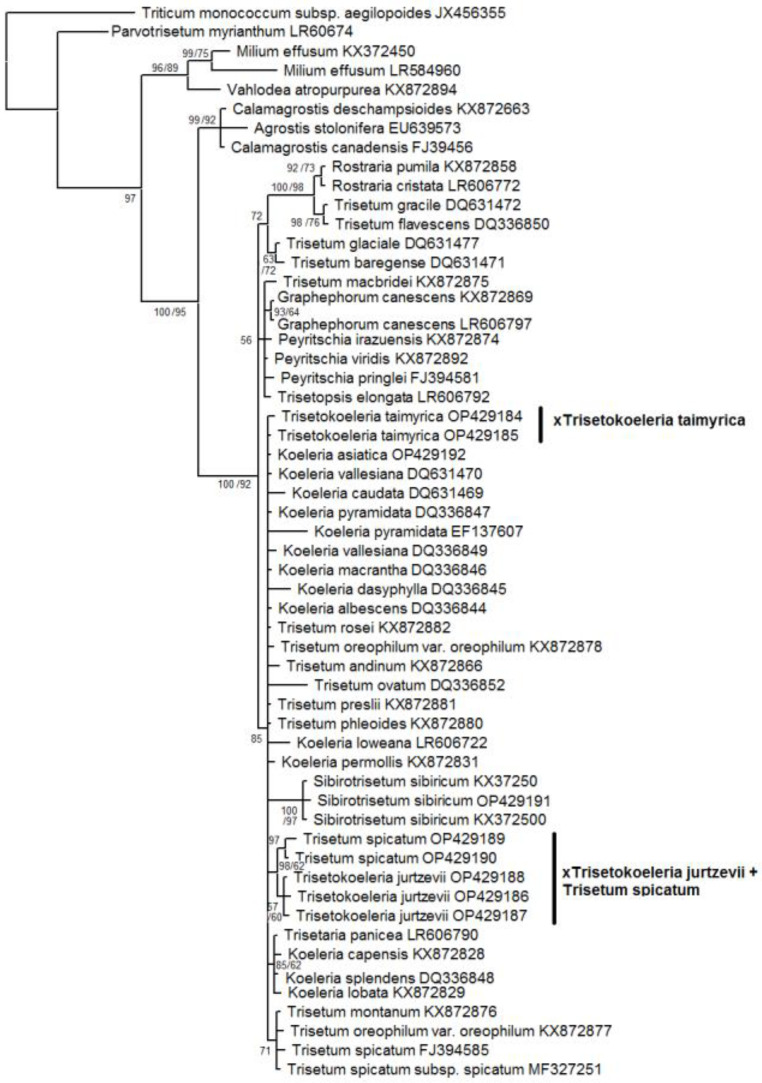
Phylogenetic tree of the genus ×*Trisetokoeleria* and related species according to the *trn*L–*trn*F sequence data. The first index on the branch is the posterior probability in Bayesian inference, the second is the bootstrap index obtained by Maximum Likelihood algorithm. When only one index is shown on the branch it is the posterior probability.

Our matrices of the data obtained by the Sanger method consisted of the following: 1159 *trn*L–*trn*F aligned positions; 702 *trn*K–*rps*16 positions; and 585 ITS1–5.8S rDNA–ITS2 aligned positions. (Tables S1–S3, Supplementary). We used only sequences of ×*Trisetokoeleria jurtzevii* and ×*T. taimyrica* in this analysis. According to the tree constructed on sequences of the chloroplast region *trn*L–*trn*F, the species of *Trisetum*, *Koeleria*, and ×*Trisetokoeleria* formed a large polytomy within the clade, comprising the taxa of the subtribe *Koeleriinae* Asch. and Graebn. s. l. (Figure 4).

The subclades in this clade corresponded to some sections of the following: *Trisetum* and *Koeleria* ((*Koeleria capensis* Nees + *K. lobata* Roem. & Schult. +*K. splendens* C.Presl + *Trisetaria panicea* (Lam.) Paunero); (*Trisetum gracile* Boiss. + *T. flavescens* (L.) P.Beauv.) + (*Rostraria pumila* (Desf.) Tzvelev + *R. cristata* (L.) Tzvelev); and (*Graphephorum canescens* (Buckley) Röser & Tkach + *T. macbridei* Hitchc.)). On this tree, ×*Trisetokoeleria jurtzevii* belonged to the clade with Altaic samples of *Trisetum spicatum* (PP = 98, BS = 62, Figure 4). We need to note that *T. spicatum* samples from Mexico (GenBank data) fall into the low-supported clade with *T. montanum* Vasey and *T. oreophilum* Louis-Marie (PP = 71, BS unsupported), and they do not group with Eurasian ones and ×*Trisetokoeleria jurtzevii*, as was recovered through analysis of the *trn*L–*trn*F sequences. *Trisetokoeleria taimyrica* occupied an uncertain position within a moderately supported clade (PP = 85, BS = 92) to which belong all *Koeleria* species, *Trisetum spicatum* with some relatives and the recently separated genus *Sibirotrisetum* Barberá, Soreng, Romasch., Quintanar & P.M.Peterson [9] (Figure 4).

**Figure 5:**
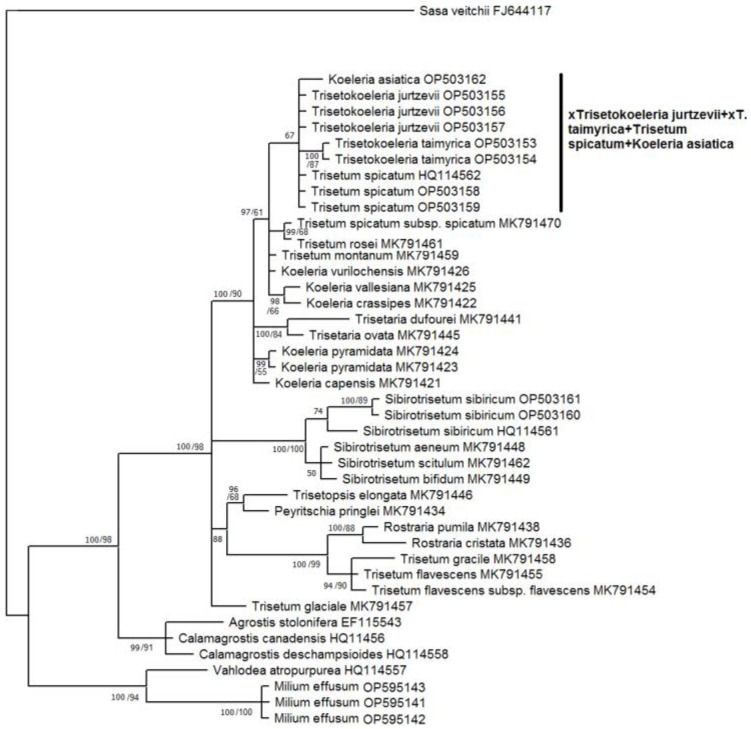
Phylogenetic tree of the genus ×*Trisetokoeleria* and related species according to the *trn*K–*rps*16 sequence data. The first index on the branch is the posterior probability in Bayesian inference, the second is the bootstrap index obtained by Maximum Likelihood algorithm. When only one index is shown on the branch it is the posterior probability.

The second studied region, *trn*K–*rps*16, provided us with a better resolution (Figure 5).

As in the previous phylogenetic scheme (Figure 4), the genus ×*Trisetokoeleria* with *Trisetum* s. l. and *Koeleria* formed the large clade, Koeleriinae. Here, nothospecies ×*Trisetokoeleria taimyrica* formed a subclade (PP = 100, BS = 87) within the low-supported clade (PP = 67, BS unsupported) with the following: Altaic samples of *Trisetum spicatum*; *T. spicatum* from Mexico; *Koeleria asiatica* (Altai Mountains); and nothospecies ×*Trisetokoeleria jurtzevii*. This subclade, in its turn, belonged to the clade highly supported in Bayesian analysis (PP = 97, BS = 61) with the Mexican samples of *T. spicatum* + *T. rosei* Scribn. and Merr.; *Koeleria vurilochensis* C.E.Calderón ex Nicora*; T. montanum*; and *Koeleria vallesiana* Asch. and Graebn. + *K. crassipes* Lange. The species of the genus *Trisetaria* Forssk., *Koeleria pyramidata* P.Beauv., and *K. capensis* formed a polytomy with this group in the strongly supported clade (PP = 100, BS = 90, Figure 5). *Sibirotrisetum* (=*Trisetum*) *sibiricum* with related species forming a strongly supported clade (PP = 100, BS = 100). However, the *T. flavescens* fell within a separate clade (PP = 88) with *T. gracile*, a species of the genus *Rostraria* and *Trisetopsis elongata* (Hochst. ex A.Richt.) Röser and A.Wölk + *Peyritschia pringlei* (Scribn.) S.D.Koch. These two clades formed a polytomy with the clade containing ×*Trisetokoeleria*, *Trisetum spicatum* and the *Koeleria* species (Figure 5).

**Figure 6:**
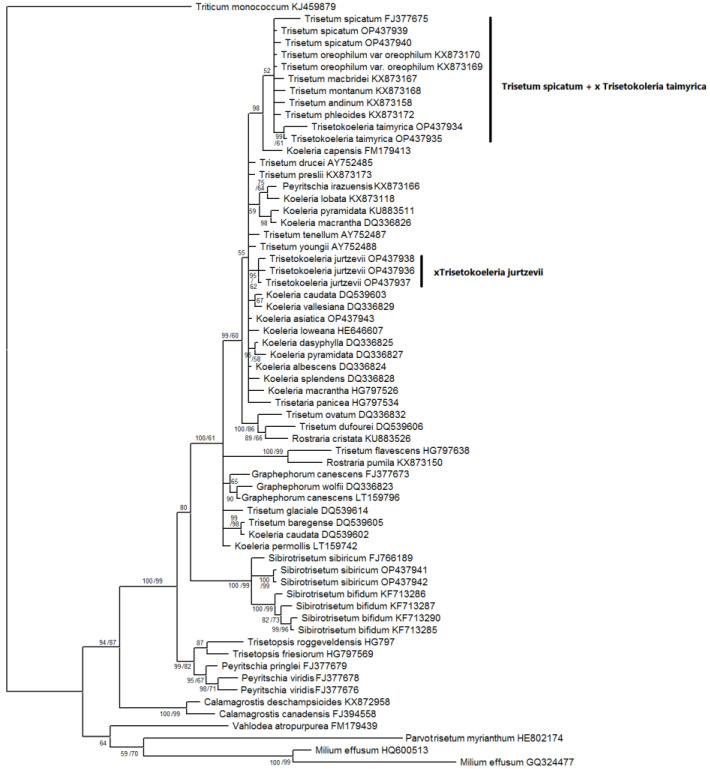
Phylogenetic tree of the genus ×*Trisetokoeleria* and related species according to the ITS sequence data. The first index on the branch is the posterior probability in Bayesian inference, the second is the bootstrap index obtained by Maximum Likelihood algorithm. When only one index is shown on the branch it is the posterior probability.

The tree constructed on ITS data (Figure 6) shows us a slightly different pattern, though the Koeleriinae clade was retained in all analyses.

Two samples of ×*T. taimyrica* formed a subclade (PP = 99, BS = 61) in the clade with the following: *Koeleria capensis*; *Trisetum phleoides* Kunth; *T. macbridei*; *T. andinum* Benth.; *T. montanum*; *T. oreophilum*; and *T. spicatum* (PP = 98, BS unsupported). The ×*Trisetokoeleria jurtzevii* samples formed a polytomy with different species of *Koeleria*, *Trisetum* (*T. tenellum* (Petrie) Allan and Zotov ex Laing and Gourlay, *T. youngii* Hook.f., *T. drucei* Edgar, and *T. preslii* (Kunth) É.Desv.), and *Trisetaria panicea*. All these species formed a highly supported subclade in the Bayesian inference (PP = 99, BS = 60) within the large clade (PP = 100, BS = 61) that also contained the following: *Trisetum flavescens + Rostraria pumila*; *Graphephorum canescens + G. wolfii* J.M.Coult.; *Trisetum glaciale* (Bory) Boiss.; *T. baregense* Laffitte & Miégev. + *Koeleria caudata*; and *K. permollis* Steud. *Sibirotrisetum sibiricum* (=*Trisetum sibiricum*) and allied *S. bifidum* (Thunb.) Barberá group was in a separate clade, which was sister to the big clade comprising all of above-mentioned species.

### Pollen Fertility Analysis

Our acetocarmine staining of pollen grains of ×*Trisetokoeleria* species and their possible progenitors gave the following results (Table 4).

The ×*T. jurtzevii* yielded completely sterile pollen, while ×*T. taimyrica* had not much more sterile pollen than the non-hybrid species, and ×*T. gorodkowii* was intermediate with 45% abortive pollen cells. Non-hybrid, possible parental species had a low proportion of abortive pollen (Table 4).

## 3. Discussion

### 3.1. Overview of the Genus

The ×*Trisetokoeleria* is an example of a hybrid genus in the grass family. The hybrid genera are quite rare among the members of the tribe Poeae s. l. and more common in the tribe Triticeae Dumort. Nevertheless, processes of hybridization occurring within the tribe Poeae s. l. are active, and they are often detected by modern molecular phylogenetic methods.

As a result of multiple reticulation, the entire *Trisetum* genus was relatively recently regarded as polyphyletic with different clades of *Trisetum* s. l. uniting with many small genera of the subtribe Koeleriinae [6,7,8,9]. In fact, this multiple division of the genus tells us that its species could be part of an introgressive–interspecies complex, a hybrid swarm [16,17,18,19,20], where morphological similarity of its members is a result of genome absorption in a series of introgressive hybridizations.

The evolution of the hybrid genus ×*Trisetokoeleria* may be related to the high latitudes of the Northern Hemisphere. Despite the fact that its possible parental species also grow together in Asian mountains, e.g., in the Altai–Sayan region, we have not seen any occurrences of hybridization between *Trisetum* and *Koeleria* (both in a narrow sense) outside the Arctic. Arctic conditions are extreme and, thus, facilitate the processes of polyploidization and hybrid speciation for adaptation and surviving [21,22]. The ×*Trisetokoeleria* species are neopolyploids, in terms of their hybrid state [23,24,25], formed rather recently and probably reproduced mostly vegetatively, at least two of them did. As we see from the pollen fertility analysis, ×*T. jurtzevii* is a sterile hybrid, and ×*T. gorodkowii* is most likely in the early stages of hybrid stabilization. However, our data on their origin showed some differences from the evolutionary hypotheses based on morphological criteria.

### 3.2. Origin of Each Species According to the Molecular Data

The nothospecies ×*T. jurtzevii* had one main ribotype that was not shared with any other of the studied species (Figure 1). It clearly differed from the main ribotype of *Trisetum spicatum* obtained in our research and ITS sequences taken from GenBank data. As the second major ribotype of ×*T. jurtzevii* was common with *Koeleria asiatica*, we could suppose that ×*T. jurtzevii* originated from the intercrossing of *K. asiatica* s. l. and some Arctic race of *Trisetum* close to *T. spicatum* affinity. From the *trn*L−*trn*F gene analysis (Figure 4) we could see that some relative of *T. spicatum* probably gave the maternal genome to ×*T. jurtzevii*. The results of the analysis of the ITS sequences (Figure 6), obtained by the Sanger method (longer region than studied by NGS), revealed that ×*T. jurtzevii* was unrelated to any other species of *Trisetum* and *Koeleria* within the common clade of most of the Koeleriinae members. This allowed us to assume not only possible post-hybridization transformation of the sequences via intergenomic conversion [26], but also significant genetic difference between Arctic and Siberian mountain *Trisetum* species. In addition, one of the ×*T. jurtzevii* ancestral taxa could probably be extinct, and the hybrid persisted for a long time via vegetative propagation. The fact that in the samples from geographically distant locations different sequences from the whole rDNA pool were amplified by the Sanger method could be the result of polyploidy of *T. spicatum* and allied species (mostly 2n = 28—[13,27,28,29,30]).

The ×*Trisetokoeleria gorodkowii* had only one main ribotype that was shared with *Trisetum spicatum* and *T. wrangelense* (the minor fraction in their genomes). According to the marker sequences obtained by NGS, there was no connection between this hybrid and *Trisetum ruprechtianum*, which most likely belonged to a separate line now named *Sibirotrisetum* [8]. The ×*T. gorodkowii* was first described as *Koeleria gorodkowii* Roshev [31], belonging to the section *Caespitosae* Domin related to *K. pyramidata* affinity. From a morphological point of view, it was more reminiscent of the species of *Koeleria* than those of *Trisetum*. Our NGS and pollen analyses results showed that ×*T. gorodkowii* was most probably a modern hybrid, having sequences close to the *Koeleria* line retained in the allopolyploid genome. However, ×*T. gorodkowii* was not related to *K. asiatica* s. l. and, therefore, might be a derivative of the *K. pyramidata* group instead. The second parental taxon of ×*T. gorodkowii* was probably some relative of *Trisetum spicatum* group (Arctic races) but not *Trisetum ruprechtianum* or any other ally of the newly separated genus *Sibirotrisetum* [8]. Marker sequences of the second parental species could be deleted from the allopolyploid genome.

The ×*Trisetokoeleria taimyrica* had one main ribotype common with *Trisetum spicatum* and *T. wrangelense.* According to NGS data, this ribotype was unrelated to the ribotypes of *T. subalpestre*. The analysis of the ITS sequences performed by the Sanger method demonstrated a similar result: ×*T. taimyrica* was close to *T. spicatum* but formed a separate subclade on the tree (Figure 6), not connected with *Koeleria asiatica*. In addition, the chloroplast sequences of the ×*T. taimyrica* group with *Trisetum spicatum* and *Koeleria asiatica* (*trn*K−*rps*16 data, Figure 5) also fell within the separate subclade. Pollen fertility in ×*T. taimyrica* was more pronounced than in ×*T. gorodkowii*; this might be due to the stabilized hybrid status of the species, allowing it to propagate in seeds as well as in vegetative clones. Recent molecular phylogenetic research placed ×*T*. *taimyrica* within the genus *Koeleria* because of the ITS and cpDNA sequence data showing close affinity of *Trisetum spicatum* to the *Koeleria* members [8]. Nevertheless, our ITS data showed more close affinity of ×*T*. *taimyrica* to the *T. spicatum* group than to the most part of the genus *Koeleria*. According to the NGS data, the main ribotype of ×*T*. *taimyrica* also belonged to the *Trisetum* group and was not closely related to the ribotypes of *Koeleria*. We tended to retain the hybrid status and generic name ×*Trisetokoeleria* for ×*T*. *taimyrica* although the hybridization in this taxon could have occurred earlier in its evolutionary history, according to the pollen staining data.

Thus, we could assume that ×*Trisetokoeleria taimyrica* was probably a stabilized introgressive hybrid between *Trisetum wrangelense* (arctic member of *T. spicatum* group) and *Koeleria asiatica* (probably even from multiple introgressions). In addition, ×*Trisetokoeleria taimyrica* had two minor ribotypes in its rDNA pool that reflected hybridization with some more distant relative (Figure 1). It was close to the ITS sequences of *Peyritschia deyeuxioides* (Kunth) Finot and *Cinnagrostis viridiflavescens* (Poir.) P.M.Peterson, Soreng, Romasch. and Barberá (GenBank data). These Central and South American species belong to the separate line including *Deyeuxia* and *Calamagrostis* Adans. genera (former *Calamagrostis* s. l., Figure 4, Figure 5 and Figure 6). Of course, we could not say with exactitude that these species formed hybrid ×*T. taimyrica*, but it seemed that some lineage of *Calamagrostis* s. l. (probably not only one species) might have been involved in the formation of this hybrid. Affinity between arctic and southern species may be an interesting fact that brings to mind the interpolar disjunction in grass evolution [32,33,34,35,36]. 

Presumably, the species carrying the genome that became the parent of ×*T. taimyrica*, related to the southern group of *Calamagrostis* s.l., could have migrated to South America at first through Beringia and, later, further along the Cordillera mountain range. We also could not exclude the possibility that this ancestral species that took place in ×*T. taimyrica* formation may have become extinct recently, because modern *Calamagrostis* and *Deyeuxia* species from boreal and arctic regions usually have ITS sequences unrelated to those of southern species.

### 3.3. Allopolyploid Parental Taxa of ×Trisetokoeleria and Problem of Delimitation of the Genera

It is important to pay attention to the possible hybrid status of the parental taxa of ×*Trisetokoeleria*. In modern classifications, based on molecular phylogenetic analyses of different nuclear and chloroplast genes of *Trisetum*, the section *Trisetaera* (Asch. and Graebn.) Honda to which *T. spicatum* belongs was moved to the genus *Koeleria* [8,20]. This decision could be supported by the similarity of some morphological characteristics of these taxa, such as dense and narrow panicles with hairy branches, and, sometimes, subequal glumes [8,37]. Nevertheless, the genus *Trisetum* (even in the broader sense, including the members of sect. *Trisetaera*), also has morphological features that are clearly different from the genus *Koeleria*. The first, and one of the main distinguishing features, is the awn on the lemma. In the genus *Trisetum* sect. *Trisetaera* the species have dorsal well-developed lemma awn usually inserted slightly below the apex [8]. The awn is bent basally to sub-basally [8]. In addition, the lemmas of *Trisetum* have two awn-like teeth on the tip. *Koeleria*, in its turn, usually has only slightly acute lemma apex without any awns. In some rare cases, the species of *Koeleria* have very short mucro right on the lemma apex. Lemma callus in the genus *Trisetum* is acute, whereas that of *Koeleria* is obtuse [13]. Molecular phylogenetic data (Figure 4, Figure 5 and Figure 6, see also [9,20]) showed multiple hybridization events in this group. For example, *Trisetum spicatum* fell into the clade with ×*Trisetokoeleria taimyrica*, *Trisetum oreophilum*, *T. montanum*, *T. phleoides*, *T. andinum*, and one *Koeleria* species, *K. capensis*, but was distant from *K. asiatica*, according to the ITS analysis (Figure 6). Chloroplast gene data (*trn*L–*trn*F), on the contrary, placed *Trisetum spicatum* from the Altai Mountains with ×*Trisetokoeleria jurtzevii* and *T. spicatum*, from Mexico (GenBank accessions), formed the separate clade with *T. montanum* and *T. oreophilum* (Figure 4). The maternal genome of *Trisetum spicatum* could be related to *Koeleria asiatica* (*trn*K–*rps*16 data), though not closely (Figure 5). All sequence data placed *T. spicatum* rather distantly from *Koeleria pyramidata*. The ITS and chloroplast sequences clearly showed the possibility of multiple introgressive hybridizations in the sect *Trisetaera*, since different samples of the same species gave different phylogenies. The ITS1 sequences, obtained by NGS, showed that the main ribotype of *Trisetum spicatum* was common to that of *T. wrangelense* and with only minor ribotype fraction of *Koeleria altaica*. We need to note that *Trisetum spicatum* was unrelated to the type species of the genus, *Trisetum flavescens*. However, in its turn, *T. flavescens* formed a clade with *Rostraria pumila*, based on both chloroplast and ITS sequences. Previous researchers did not unite *Trisetum* with annual grasses of the genus *Rostraria*, pointing to the developed hybridization within the subtribe Koeleriinae [9,20]. Of course, multiple introgression events could take place in our case, in the clade with *Trisetum* sect. *Trisetaera*, other *Trisetum* species (*T. drucei*, *T. preslii*), and *Trisetaria* Forssk. We see here a phylogenetic picture that is a reminder of intergeneric hybrid speciation in the tribe Triticeae. For example, the genus *Elymus* L. s. l. originated from the intercrossing of St, H, and Y-genome bearers belonging to different genera [38,39]. New taxonomic treatment of *Elymus* and its relatives proposed generic names based on the genome combinations that the species has: *Elymus* s. str. (StH), *Roegneria* K.Koch (StY), *Campeiostachys* Drobov (StYH), etc. [40,41]. At the same time, genome combinations in the subtribe Koeleriinae are unknown to us yet. Thus, we cannot estimate contribution of each genome in the formation of the allopolyploid *Trisetum* species as well as in its allies. For our convenience, and taking into account multiple reticulation events between the species and in main lineages of the subtribe Koeleriinae [20], we preferred to retain the previous classification which separates the most part of *Trisetum* (including sect. *Trisetaera*) and *Koeleria*. We also distinguished the specific clade representing the genus *Sibirotrisetum*. We need to mention that morphological traits in this case more or less reflected the genome combinations.

As can be seen, *Koeleria asiatica*, as well as *Trisetum spicatum* (sect. *Trisetaera*) and *T. subalpestre* (sect. *Agrostidea* Prob. close to the section *Trisetaera*—[8]), were most probably of introgressive hybrid origin (Figure 1, Figure 2 and Figure 3). *K. asiatica* had the main ribotype T2 shared with *T. subalpestre* (the second major ribotype), but also in *K. asiatica* there was one unique ribotype Ka that was far less frequent in the genome (7%, Figure 1). In addition, *K. asiatica* had a minor fraction of T1 ribotype that was characteristic for *Trisetum spicatum* and *T. wrangelense*. Thus, we could assume that *K. asiatica* was an allopolyploid from intercrossing of *T. subalpestre* and some *Koeleria* species, probably with little participation of *T. spicatum s.* l. According to our analysis, *K. asiatica* certainly belonged to the eupolyploid (mesopolyploid) stage of polyploid evolution when the karyotype in meiosis behaves as a classic diploid without any failures, but the parental genomes can still be separated cytogenetically [24].

## 4. Conclusions

Our NGS data, together with chloroplast and nuclear sequences obtained by the Sanger method, gave us some new and important information about the origin and possible parental taxa of three species of nothogenus ×*Trisetokoeleria* from the Poeae tribe. This information partly contradicted morphological assumptions. However, we can also say that the NGS data helped clarify some previous hypotheses built on previous molecular phylogenetic results (because the nuclear sequences obtained by the Sanger method may have lower ability in distinguishing nucleotide positions, due to conversion in allopolyploid genomes). Looking at the polyploid members of *Trisetum*/*Koeleria* affinity we needed to take into account possible multiple cases of introgression. Nevertheless, we tended to retain the previous generic names for the most part of this tribe. All species of these taxa may be intergeneric hybrids, and the morphological features used in delimitation of generic borders depended on which proportion of the parental genomes was present in them. Our methods of molecular phylogenetic analysis, along with analysis of pollen fertility, allowed us to successfully assume the order of the formation of nothospecies. The ×*T. jurtzevii* is the most recent sterile hybrid, originated from *Koeleria asiatica* and some northern species of *Trisetum* (not *T. spicatum* s. str.), whereas ×*T. gorodkowii* is a hybrid in the stage of primary stabilization, probably formed not long ago from some *Trisetum* species unrelated to *T. ruprechtianum*, and ×*T. taimyrica* is a stabilized ancient hybrid species, originated from *Koeleria asiatica* and *Trisetum wrangelense*.

## 5. Materials and Methods

### 5.1. Molecular Phylogenetic Analysis

In our study, we used 441 sequences of 18S–ITS1–5.8S rDNA obtained via NGS from eight species: ×*Trisetokoelria gorodkowii*, ×*T. jurtzevii*, ×*T. taimyrica* and their putative ancestors: *Koeleria asiatica*, *Trisetum ruprechtianum*, *T. spicatum*, *T. subalpestre*, *T. wrangelense* (V.V.Petrovsky) Prob. All our samples were taken from the Herbarium of Komarov Botanical Institute (LE). Information on these species with their accession numbers and ribotypes is presented in Table 1.

We sequenced the ITS1–5.8S rDNA–ITS2 region, *trn*L–*trn*F (including *trn*L gene, *trn*L intron, and *trn*L–*trn*F intergenic spacer) and *trn*K–*rps*16 from eight samples of five species: ×*Trisetokoeleria jurtzevii*, ×*T. taimyrica*, *Koeleria asiatica*, *Sibirotrisetum sibiricum* (=*Trisetum sibiricum*), *T. spicatum* using the Sanger method. GenBank numbers of ITS1–5.8S rDNA–ITS2, *trn*L–*trn*F, *trn*K–*rps*16 are given in Table 2.

Genomic DNA was extracted from seeds using a Qiagen Plant Mini Kit (Qiagen Inc., Hilden, Germany), according to the instruction manual. NGS was carried out at the Center for Shared Use “Genomic Technologies, Proteomics and Cell Biology” of the All-Russian Research Institute of Agricultural Microbiology on an Illumina Platform MiSeq. We used 15 µL of PCR mix containing 0.5–1 unit of activity of Q5^®^ High-Fidelity DNA Polymerase (NEB, Ipswich, MA, USA), 5 pM of forward and reverse primers, 10 ng of DNA template, and 2 nM of each dNTP (Life Technologies, ThermoScientific, Waltham, MA, USA). The PCR was carried out using primers ITS 1P [42] and ITS 2 [43] under the following parameters: initial denaturation 94 °C for 1 min, followed by 25 cycles of 94 °C for 30 s, 55 °C for 30 s, 72 °C for 30 s, and a final elongation for 5 min. PCR products were purified using AMPureXP (Beckman Coulter, Indianapolis, IN, USA). Further preparation of the libraries was carried out in accordance with the manufacturer’s MiSeq Reagent Kit Preparation Guide (Illumina) (http://web.uri.edu/gsc/files/16s-metagenomic-library-prep-guide-15044223-b.pdf (accessed on 11 May 2020)). The libraries were sequenced, according to the manufacturer’s instructions, on an Illumina MiSeq instrument (Illumina, San Diego, CA, USA) using a MiSeq^®^ ReagentKit v. 3 (600 cycle) with pair-end reading (2 × 300 n). The obtained pool of raw sequences was trimmed with the aid of Trimmomatic [44] included in Unipro Ugene [45] using the following parameters: PE reads; sliding window trimming with size 4 and quality threshold 12; and minimal read length 130. Then paired sequences were combined using the program fastq-join [46], dereplicated and sorted by vsearch 2.7.1 [47]. The resulting sequences formed ribotypes in the whole pool of genomic rDNA; they were sorted according to their frequency. For our analysis, we established a threshold of 20 reads per pool of rDNA. The sequences were aligned using MEGA X [48]; a haplotype network was built in TCS 2.1 [49] and visualized in TCS BU [50]. Obtained ribotypes were used for the neighbor-joining tree constructed in MEGA X [48], by a maximum likelihood algorithm with the parameters GTR + G. We built a neighbor net of the ribotypes using SplitsTree 4.18 [51]. For this network, we set the threshold of 30 reads per the whole pool.

The marker region ITS1–5.8S rRNA gene–ITS2 was amplified using the universal primers ITS 1P [42] and ITS 4 [43]. The amplification parameters were: one cycle of 95 °C for 5 min; 35 cycles: 95 °C for 40 s; 52–56 °C for 40 s; 72 °C for 40 s; and final elongation 72 °C for 10 min. The chloroplast region trnL–trnF was amplified with the primers tabC, tabD, tabE and tabF [52]. Amplification parameters, when working with primer pairs C and D and E and F, were as for ITS amplification, but when using only external pair C and F were as follows: one cycle of 95 °C for 5 min; 35 cycles: 95 °C for 1 min; 52–56 °C for 1 min 10 s; 72 °C for 1 min 10 s; and final elongation 72 °C for 10 min. Region trnK–rps16 was amplified using primers rps16−4547mod [53] and trnK5’r [54] following the same parameters as for the ITS fragment. The sequencing was performed according to the standard protocol provided with a BigDyeTM Terminator Kit ver. 3.1 set of reagents on the sequencer ABI PRIZM 3100 sequencer at the Center for the collective use of scientific equipment “Cellular and molecular technologies for the study of plants and fungi” of the Komarov Botanical Institute, St. Petersburg. Chromatograms were analyzed with Chromas Lite version 2.01(Technelysium co.) and then the sequences were aligned with the aid of the Muscle algorithm [55] included in MEGA X [48].

Additionally, we used 42 sequences of *trn*L–*trn*F (including *trn*L gene, *trn*L intron, and *trn*L–*trn*F intergenic spacer), 32 sequences of *trn*K–*rps*16, and 54 sequences of ITS1–5.8S rDNA–ITS2 region, from GenBank database (https://www.ncbi.nlm.nih.gov/nuccore/?term (accessed on 11 May 2020)) along with our data (Supplementary, Tables S1–S3). Each dataset was analyzed separately because of the possible hybridization events resulting in different schemes by chloroplast and nuclear gene data. Appropriate evolutionary models were computed with MEGA X [48]. Indels were coded with SeqState 1.4.1 [40] and included into the alignment file as binary data (“restriction” option). Bayesian inference was performed by Mr. Bayes 3.2.2 [41] using GTR + I + G for ITS dataset, T92 + G for *trn*L–*trn*F and T92 for *trn*K–*rps*16, under the following conditions: 1–1.5 million generations; sampling trees every 100 generations; and the first 25% trees were discarded as burn-in. Maximum likelihood analysis was conducted using the same models with 1000 bootstrap replications using MEGA X [48]. A clade with 100–90% of posterior probability (PP) and bootstrap index (BS) was treated as strongly supported; 89–70% as moderately supported; and 50–69% as weakly supported. Nodes with indices below 50 were treated as unsupported.

### 5.2. Pollen Fertility Detection

In addition, to detect the hybrid status of the species we performed pollen fertility analysis for *Trisetum spicatum*, *T. ruprechtianum*, *Koeleria asiatica*, ×*Trisetokoeleria jurtzevii*, ×*T. gorodkowii*, ×*T. taimyrica.* Three or four anthers were taken from the herbarium material and placed in an Eppendorf tube with 200−250 µL of 45% acetic acid. The required degree of maceration was achieved within 1 h. Then each anther was placed on a glass slide, 50 μL of 10% acetocarmine solution was added, the anther was covered with a coverslip and slightly heated. After that, the material was distributed under the coverslip by lightly tapping with a wooden stick. Then, under a microscope, colored and uncolored pollen grains were counted, taking into account a total of at least 1000 pieces (lens 10×). Unstained pollen grains without contents or slightly colored and deformed ones were considered abortive, while uniformly intensely colored and undeformed ones were considered conditionally fertile. Temporary preparations were also photographed using 10× and 40× objectives.

## Figures and Tables

**Table 1:** Summary of the possible parental taxa and three Trisetokoeleria species used for next-generation sequencing in the present study and their ribotypes. Chromosome numbers are given according to [13].

Species	Sample ID	2n	Country of Origin	Collected by	Accession Number in Genbank Database	Number of Accessions	Total Number of Reads	Ribotype Number in Figure 1	Ribotype Symbol	Number of Reads	% from the Total Number of the Reads
*Trisetum spicatum*	L1	28	Russian Federation: Russia: Yakutia,	S.V. Chinenko	OP557123–OP557200	78	21,799	1	T1	16,839	77
			Anabarsky District					3	T2	50	0.2
								7	T5	18	0.08
*Trisetum subalpestre*	L13	28	Russian Federation: Chukotka	A.A. Korobkov, B.A. Yurtzev	OP557201–OP557245	45	26,464	2	Ts	12,538	47
								3	T2	8685	33
*Trisetum wrangelense*	M3	28	Russian Federation: Chukotka,	B. Yurtzev, T. Polozova	OP557389–OP557522	134	27,647	1	T1	21,565	78
			Wrangel Island					3	T2	50	0.1
								7	T5	12	0.04
*Trisetum ruprechtianum*	M2	14	Russian Federation: Chukotka	V.V. Petrovsky, T.V. Plieva	OP557327–OP557388	62	12,740	4	T3	2548	20
								5	T4	1126	9
*Koeleria asiatica*	G18	14, 28	Russian Federation: Yakutia,	T.M. Koroleva	OP557523–OP557563	41	22,341	3	T2	12,064	54
			Anabarsky District					6	Ka	1505	7
								1	T1	1085	5
×*Trisetokoeleria gorodkowii*	G19	N/A	Russian Federation: Yakutia	A.A. Korobkov, T.M. Koroleva	OP557281–OP557290	10	12,048	7	T5	3492	29
×*Trisetokoeleria jurtzevii*	G16	N/A	Russian Federation: Yakutia	P.A. Gogoleva, T.M. Koroleva	OP557246–OP557280	35	14,620	8	Tj	7090	48
								3	T2	2824	19
×*Trisetokoeleria taimyrica*	G15	N/A	Russian Federation: Yakutia	P.A. Gogoleva, T.M. Koroleva	OP557291–OP557326	36	19,245	1	T1	14,574	76

**Table 2:** Sequences obtained in the present study by the Sanger method and their numbers in GenBank.

Species	Country of Origin	Collected by	Genbank Number, trnL-trnF	Genbank Number, trnK-rps16	Genbank Number, ITS
×*Trisetokoeleria jurtzevii*	Russia: Yakutia	P.A. Gogoleva, T.M. Koroleva	OP429186	OP503155	OP437936
×*Trisetokoeleria jurtzevii*	Russia: Yakutia	P.A. Gogoleva, T.M. Koroleva	OP429187	OP503156	OP437937
×*Trisetokoeleria jurtzevii*	Russia: Yakutia	T.M. Koroleva	OP429188	OP503157	OP437938
×*Trisetokoeleria taimyrica*	Russia: Yakutia	P.A. Gogoleva, T.M. Koroleva	OP429184	OP503153	OP437934
×*Trisetokoeleria taimyrica*	Russia: Yakutia	P.A. Gogoleva, T.M. Koroleva	OP429185	OP503154	OP437935
*Sibirotrisetum sibiricum*	Russia: Yakutia	M. Telyatnikov		OP503160	OP437941
*Sibirotrisetum sibiricum*	Russia: Yakutia	S.V. Chinenko	OP429191	OP503161	OP437942
*Trisetum spicatum*	Russia: Yakutia	S.V. Chinenko	OP429189	OP503158	OP437939
*Trisetum spicatum*	Russia: Yakutia, Anabarsky District	S.V. Chinenko	OP429190	OP503159	OP437940
*Koeleria asiatica*	Russia: Yakutia, Anabarsky District	T.M. Koroleva	OP429192	OP503162	OP437943

**Table 3:** Primary structure of the major ribotypes obtained by NGS. The minor ribotypes (55-G15 and 14-G15(3)) are also provided, representing distant hybridization. As the reference sequence we took the main ribotype of *Trisetum spicatum*—possible progenitor of ×*Trisetokoeleria* species. D is a deletion.

		1	1	1	1	1	1	1	1	1	1	1	2	2	2	2	2	2	2	2	3
	9	0	1	1	4	4	4	5	5	6	7	7	2	2	4	4	5	5	6	8	3
	7	9	4	6	0	7	9	0	1	8	4	9	7	9	4	8	1	5	0	1	1
T1	G	C	C	C	G	A	A	A	C	G	T	G	A	G	T	T	C	A	G	C	C
Ts	A	.	.	.	.	.	.	.	.	.	.	.	C	.	.	.	.	.	.	.	.
T2	.	.	.	.	.	.	.	.	.	.	.	.	C	.	.	.	.	.	.	.	.
T3	.	.	T	.	C	T	.	.	.	.	.	.	T	.	C	.	T	C	.	.	.
T4	.	.	T	.	C	T	.	.	.	.	A	.	T	A	C	.	T	C	.	.	.
Ka	.	.	.	.	.	.	D	D	T	.	.	.	C	.	.	.	.	.	.	T	.
T5	.	.	.	.	.	.	.	.	.	A	.	.	.	.	.	.	.	.	.	.	.
Tj	.	.	.	T	.	.	.	.	.	.	.	A	C	.	.	.	.	.	.	.	.
55-G15	.	.	.	.	.	.	.	.	.	.	.	.	T	.	.	C	.	.	C	.	.
14-G15(3)	.	T	T	.	.	.	.	.	.	.	.	.	T	.	.	C	.	.	C	.	.
Peyritschia deyeuxioides FJ377668	.	.	.	.	.	.	.	.	.	.	.	.	T	.	.	C	.	.	C	.	.
Cinnagrostis viridiflavescens KX873106	.	.	.	.	.	.	.	.	.	.	.	.	T	.	.	C	.	.	C	.	T

**Table 4:** Pollen fertility of the species of hybrid genus ×*Trisetokoeleria* and its putative parental taxa.

Species	Total	Stained	Abortive	Abortive Pollen Percent
*Trisetum spicatum*	1042	896	146	14.0%
*Trisetum ruprechtianum*	1007	886	121	12.0%
*Koeleria asiatica*	1005	932	74	7.4%
×*T. jurtzevii*	1000	0	1000	100%
×*T. gorodkowii*	1140	627	513	45.0%
×*T. taimyrica*	1080	885	195	18.1%

## Supplementary Materials

All supplementary materials are available on https://www.mdpi.com/article/10.3390/plants11243533/s1. Table S1. *Trn*L–*trn*F sequences used in our analysis. Genbank data and sequences obtained in our research are given. Table S2. *Trn*K–*rps*16 sequences used in our analysis. Genbank data and sequences obtained in our research are given. Table S3. ITS sequences used in our analysis. Genbank data and sequences obtained in our research are given.
